# Trajectory Tracking Control Method for Omnidirectional Mobile Robot Based on Self-Organizing Fuzzy Neural Network and Preview Strategy

**DOI:** 10.3390/e25020248

**Published:** 2023-01-30

**Authors:** Tao Zhao, Peng Qin, Yuzhong Zhong

**Affiliations:** College of Electrical Engineering, Sichuan University, Chengdu 610065, China

**Keywords:** trajectory tracking, self-organizing, preview strategy, omnidirectional mobile robot

## Abstract

This paper proposes a new trajectory tracking control scheme for the four mecanums wheel omnidirectional mobile robot (FM-OMR). Considering the influence of uncertainty on tracking accuracy, a self-organizing fuzzy neural network approximator (SOT1FNNA) is proposed to estimate the uncertainty. In particular, since the structure of traditional approximation network is preset, it will cause problems such as input constraints and rule redundancy, resulting in low adaptability of the controller. Therefore, a self-organizing algorithm including rule growth and local access is designed according to the tracking control requirements of omnidirectional mobile robots. In addition, a preview strategy (PS) based on Bezier curve trajectory re-planning is proposed to solve the problem of tracking curve instability caused by the lag of tracking starting point. Finally, the simulation verifies the effectiveness of this method in tracking and trajectory starting point optimization.

## 1. Introduction

FM-OMR is a kind of robot that can realize omnidirectional movement. Its biggest feature is that it can control the longitudinal and transverse speed at the same time. With this advantage, the robot can be used in many situations. With the diversification and complexity of tasks, higher requirements are put forward for trajectory tracking.

Considering that factors such as imprecise modeling and external disturbances will introduce uncertainty to the system and affect the performance of trajectory tracking, researchers have proposed some methods to deal with uncertainty. One is to enhance the robustness of the controller, including robust control [1], sliding mode control [2,3,4], fuzzy pid [5], and so on. In Ref. [1], an adaptive robust control method is proposed for a three wheeled omnidirectional mobile robot, which includes a nominal controller based on Udwadia Kalaba theory and adaptive parameter adjustment. In Ref. [2], an adaptive terminal sliding mode control scheme is proposed for an omnidirectional mobile robot with suction cups. In Ref. [5], a controller combining PID and fast terminal sliding mode is proposed, and the error limit is estimated by fuzzy system. The other is to estimate and compensate the uncertainty through observers or approximators. Including disturbance observer [6,7], ADRC [8,9,10], neural network [11,12], etc. In Ref. [6], a sliding mode observer is set in the feedback channel to overcome the influence of feedback channel uncertainty. In Ref. [8], an ADRC framework based on the physical structure of Euler Lagrangian (EL) system is proposed. In addition to satisfying the suppression of external and internal disturbances, the design of the controller is also physically interpretable. In Ref. [10], an ADRC is used in the master slave robot system. In Ref. [11], the neural network is applied to approximate the uncertainty in the system.

Studies [13,14,15] have revealed that the fuzzy system could not only describe uncertainty, but also approximate uncertainty. In Ref. [15], an interpretable fuzzy system based on multi-objective optimization is proposed, which makes it possible to deal with uncertainty. In Ref. [16], a control method based on fuzzy system modeling and fuzzy neural network approximation is proposed. For the fuzzy neural network, the input determines the range of action. However, because the range of uncertainty is not measurable, it is difficult to accurately preset the input range of the network, and a large preset range may lead to excess rules. Therefore, the network needs to have the ability of self-organizing. In order to solve this problem, scholars have also put forward some methods [17,18,19,20,21,22]. In Ref. [17], a hierarchical self-organizing fuzzy system based on an encoder is proposed. In Ref. [18], a risk function based on tracking error and estimation error is proposed as the index of network structure adjustment. In Ref.  [22], the distance between the input and the center of the fuzzy set is used as an indicator for the increase of network rules.

Inspired by the above discussion, the research motivation of this paper is to propose a new trajectory tracking control method with fuzzy neural network for FM-OMR. The contributions and advantages of the proposed method are summarized as follows.

(1) The idea of self-organizing is introduced into the trajectory tracking control of omnidirectional mobile robots. The algorithm can dynamically generate network rules according to the motion state of the robot. It can expand the input range of the network while ensuring the independence of the rules, which not only improves the adaptability of the network but also ensures the simplification of the rules. In addition, the algorithm uses the principle of local access and sets the activation intensity threshold to limit the access range, which can not only ensure the integrity of the rule base, but also effectively reduce the number of rule accesses and reduce the computational cost.

(2) A PS based on Bezier curve is proposed. By re-planning the trajectory, the problem of tracking deformity caused by the lag of track starting point is solved, which optimizes the initial tracking process and improves the rationality of the tracking behavior.

(3) The simulation results verify the effectiveness of tracking control and the role of the self-organizing algorithm in rule growth and access control, as well as the optimization role of PS in the initial stage of tracking.

The structure of this paper is as follows. Models and problems are introduced in Section 2. The design details of the control scheme are shown in Section 3. Section 4 shows the simulation results. Finally, the conclusion is presented in Section 5.

## 2. Models and Problem

**Figure 1:**
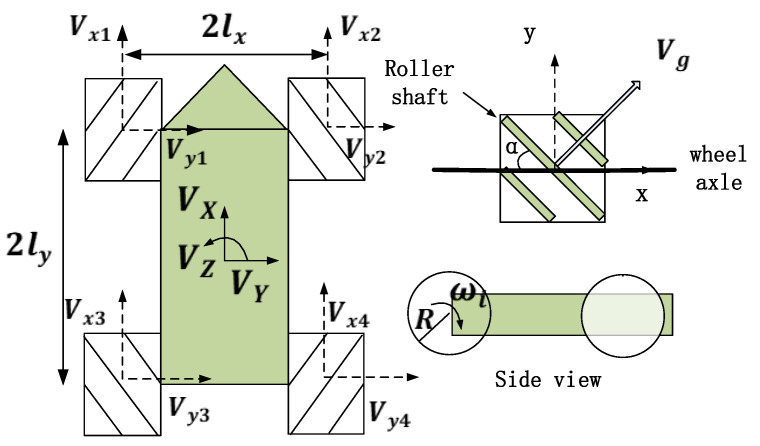
Structure diagram of FM-OMR.

The structure diagram of the FM-OMR in this paper is shown in Figure 1. The arrangement of wheels is symmetrical. Each mecanum wheel has an angle of $\alpha_{g}$ between the roller shaft and the wheel shaft. Based on Ref. [23], the kinematic model is as follows.

(1) $$\left[\begin{array}{c} \omega_{1}\\ \omega_{2}\\ \omega_{3}\\ \omega_{4} \end{array}\right]=\frac{1}{R}\left[\begin{array}{ccc} 1 & 1 & -\left(l_{x}+l_{y}\right)\\ -1 & 1 & l_{x}+l_{y}\\ 1 & 1 & l_{x}+l_{y}\\ -1 & 1 & -\left(l_{x}+l_{y}\right) \end{array}\right]\left[\begin{array}{c} V_{X}\\ V_{Y}\\ V_{Z} \end{array}\right]$$

where $\omega_{i}$ is the angular velocity, *R* is the radius of wheel, $l_{x}$ is the track width, $l_{y}$ is the wheelbase, and $\left(V_{X},V_{Y},V_{Z}\right)$ is the velocity of robot. The inverse solution is (2).

(2) $$\left[\begin{array}{c} V_{X}\\ V_{Y}\\ V_{Z} \end{array}\right]=\frac{R}{4}\left[\begin{array}{cc} \begin{array}{cc} \begin{array}{c} 1\\ 1\\ \frac{-1}{l_{x}+l_{y}} \end{array} & \begin{array}{c} -1\\ 1\\ \frac{1}{l_{x}+l_{y}} \end{array} \end{array} & \begin{array}{cc} \begin{array}{c} 1\\ 1\\ \frac{1}{l_{x}+l_{y}} \end{array} & \begin{array}{c} -1\\ 1\\ \frac{-1}{l_{x}+l_{y}} \end{array} \end{array} \end{array}\right]\left[\begin{array}{c} \omega_{1}\\ \omega_{2}\\ \omega_{3}\\ \omega_{4} \end{array}\right]$$

The kinematic model only reflects the relationship between velocity and angular velocity. The dynamic model is also considered for accurate trajectory tracking control. The following ideal dynamic models can be obtained from Ref. [24]. The definition of the parameters in (3) is presented in Table 1.

(3) $$\left\{\begin{array}{c} M\dot{\omega }+D_{\omega }\omega =\tau \\ M=\left[\begin{array}{cccc} \Upsilon +\Xi +I_{w} & -\Upsilon & -\Upsilon +\Xi & \Upsilon \\ -\Upsilon & \Upsilon +\Xi +I_{w} & \Upsilon & -\Upsilon +\Xi \\ -\Upsilon +\Xi & \Upsilon & \Upsilon +\Xi +I_{w} & -\Upsilon \\ \Upsilon & -\Upsilon +\Xi & -\Upsilon & \Upsilon +\Xi +I_{w} \end{array}\right]\\ D_{\omega }=diag\left(\mu_{1},\mu_{2},\mu_{3},\mu_{4}\right),\mu_{i}\geq 0\\ {\tau =}_{\;}{\left[\tau_{1},\tau_{2},\tau_{3},\tau_{4}\right]}^{T}\\ \Upsilon =\frac{I_{z}R^{2}}{16{\left(l_{x}+l_{y}\right)}^{2}}\;\;,\Xi =\frac{m_{r}R^{2}}{8} \end{array}\right.$$

Since there may be uncertainty in the system, the dynamic model with uncertainty is (4)

(4) $$M\dot{\omega }+D_{\omega }\omega +F\left(\omega \right)=\tau$$

where F(ω) is used to describe the uncertainty of the system. The core of this paper is to propose a trajectory tracking control method based on the above model to overcome the uncertainty in the model and achieve FM-OMR trajectory tracking.

## 3. Control Method Design

### 3.1. Overall Structure of Method

**Figure 2:**
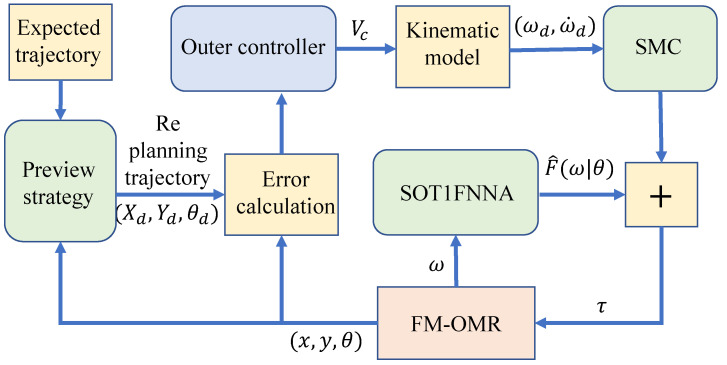
Control structure diagram.

To achieve position tracking, the proposed control method in this paper adopts a double loop control structure, which consists of an outer loop controller, an inner loop controller, and SOT1FNNA. The overall structure is shown in Figure 2.

It can be seen from the diagram, after a given desired trajectory, that the system first plans the trajectory according to the preview strategy, then obtains the desired angular velocity based on the position error and the outer loop controller. The inner loop sliding mode controller and SOT1FNNA work together to track the angular speed of the robot and finally achieve the track tracking.

### 3.2. Inner Loop Controller

The inner loop controller is mainly used to track the speed signal, which can be converted into tracking the angular speed of wheels. The mathematical expression is (5). Where $\omega_{d}$ is the desired angular velocity, ω is the actual angular velocity.

(5) $$\lim_{t\to \infty }\omega_{d}-\omega =0$$

Set the sliding surface to $s=\omega_{d}-\omega$. (6) can be obtained according to sliding condition $\dot{s}=0$.

(6) $$M{\dot{\omega }}_{d}+D_{\omega }\omega +F\left(\omega \right)-\tau =0$$

Then, the equivalent control law is (7).

(7) $$\begin{array}{c} \tau =M{\dot{\omega }}_{d}+D_{\omega }\omega +F\left(\omega \right) \end{array}$$

According to arrival condition $s\dot{s}\leq 0$, the expression of the inner loop controller is (8).

(8) $$\begin{array}{c} \tau =M{\dot{\omega }}_{d}+D_{\omega }\omega +F\left(\omega \right)+Hs \end{array}$$

### 3.3. Design of SOT1FNNA

**Figure 3:**
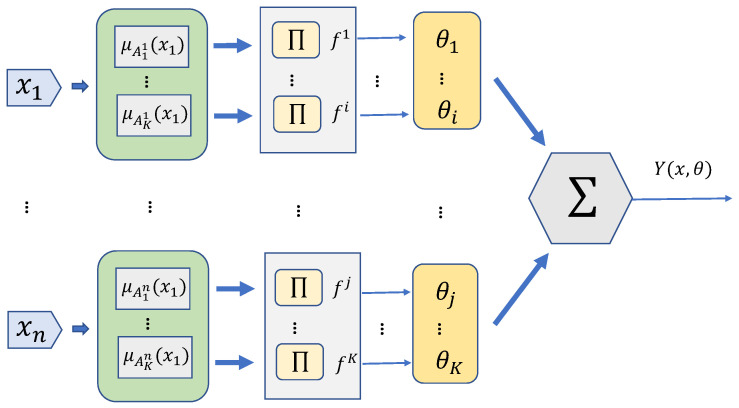
Structure of SOT1FNNA.

Since (8) contains uncertainties, it is necessary to construct SOT1FNNA for approximation and compensation. SOT1FNNA is constructed based on rule semantics [25], and each rule can be expressed as (9).

(9) $$\begin{array}{cc} \mathrm{Rule}\;i: & \mathrm{If}\;x_{1}\;\mathrm{is}\;A_{i}^{1},\ldots ,\;\mathrm{and}\;x_{n}\;\mathrm{is}\;A_{i}^{n}\\ & \mathrm{Then}\;y\;\mathrm{is}\;\theta_{i},i=1,\cdots ,K \end{array}$$

where $x_{j},j=1\ldots n$ represents the input variable of the network, and ${A_{i}}^{j},i=1\ldots K,j=1\ldots n$ is the antecedent fuzzy set. $\theta_{i}$ is the output parameters. The form of fuzzy set is shown in (10).

(10) $$\mu_{j}^{i}\left(x_{j}\right)=\exp\left\{{\left(\frac{x_{j}-m_{j}^{i}}{\sigma_{j}^{i}}\right)}^{2}\right\}$$

where $m_{j}^{i}$ is the center of the fuzzy set, $\sigma_{j}^{i}$ is the width of fuzzy set. The output of the network can be expressed as (11)

(11) $$y=\frac{\sum_{k=1}^{N}f^{k}\theta_{k}}{\sum_{k=1}^{N}f^{k}}=\sum_{k=1}^{N}{\theta_{k}\zeta }^{k}=\theta \zeta \left(x\right)$$

where $\zeta \left(x\right)=\left[\zeta^{1},\zeta^{2},\ldots ,\zeta^{k}\right]$ is the fuzzy basis vector. The structure of SOT1FNNA is shown in Figure 3.

In many applications of fuzzy neural network approximators, the network structure is preset, which may lead to some problems. When the network input exceeds the preset range, the network will not output the correct result. In addition, in some cases, the preset rules may be contradictory and cannot be activated, resulting in rule redundancy. Based on the above problems, a self-organization algorithm is proposed. The definition of variables in the algorithm is shown in Table 2, and the algorithm flow is shown in Algorithm 1.
**Algorithm 1** Self-organizing algorithm.**Require:**$x_{1}$,$Mt^{x_{1}}$,$Mt1^{x_{1}}$,$A_{i}$,RB,Rtab1,$N_{x_{1}}$. Initialize the center of $A_{new}$ as $x_{1}+0.5$ and the   width of $A_{new}$ as 5.   **for** i=1 to $N_{x_{1}}$ **do**     Compute membership degree     $\mu_{A_{i}^{1}}\left(x_{1}\right)=\exp\left\{{\left(\frac{x_{1}-m_{j}^{i}}{\sigma_{j}^{i}}\right)}^{2}\right\}$   **end for**   Compute $M_{max}^{x_{1}}=max\left(\mu_{A_{i}^{1}}\left(x_{1}\right)\right)=\mu_{A_{m}}\left(x_{1}\right)$   **if** $M_{max}^{x_{1}}<Mt^{x_{1}}$ **then**     generate new rule     $\left(if\;x_{1}=A_{new},\;then\;y=\theta_{new}\right)$   **end if**   **for** i=1 to $N_{x_{1}}$ **do**     **if** $\mu_{A_{i}^{1}}\left(x_{1}\right)>Mt1^{x_{1}}$ **then**       insert *i* to Rtab1     **end if****end for****for***i* in Rtab1 **do**     access the rule   **end for**

The algorithm includes rule growth and local access. The rule growth is mainly used to solve the problem of limited network input. The matching degree of fuzzy antecedents can be used to judge whether there are appropriate rule correspondences, which can be used as the basis for rule generation. Considering the repeatability of the robot state, the rules generated in the past should be retained, so there is no need to consider deleting rules. In addition, the growth of rules will bring greater computational pressure. Local access can effectively reduce the active rules by restricting the access scope, thereby reducing the amount of computation. According to Algorithm 1, the rule base is complete only in the worst case, and according to the local access principle, the rules activated each time are limited, so the access complexity is O(1).

For the FM-OMR, we can construct SOT1FNNA as $\hat{F}\left(\omega |\theta \right)$. The adaptive law can be designed as (12).

(12) $$\dot{\hat{\theta }}=\frac{1}{\Gamma_{r}}s\zeta \left(\omega \right)$$

To sum up, the final internal loop controller is (13)

(13) $$\tau =M{\dot{\omega }}_{d}+D_{\omega }\omega +\hat{F}\left(\omega |\theta \right)+Hs$$

where *H* is the positive definite adjustable diagonal matrix.

### 3.4. Outer Loop Controller

**Figure 4:**
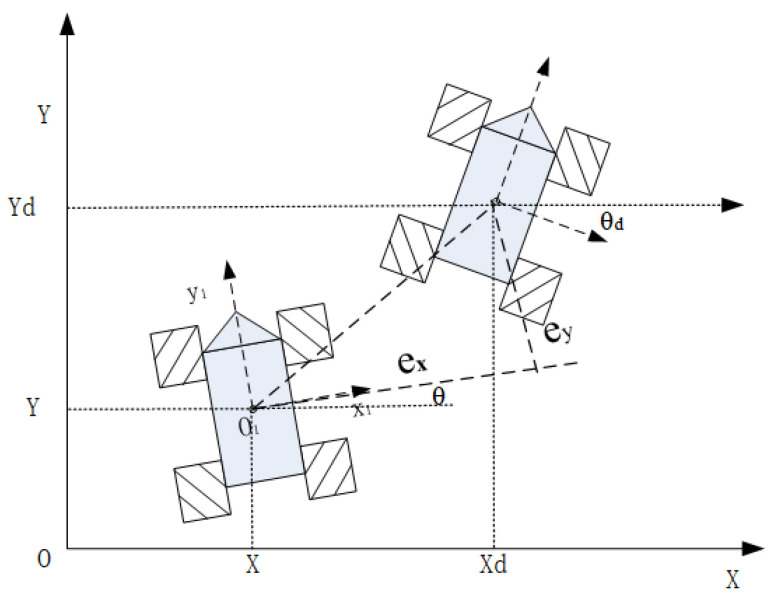
Tracking error.

The outer loop controller is a feed forward proportional controller. From Figure 4, the errors of inertial coordinate system and body coordinate system are (14) and (15).

(14) $$E=\left[\begin{array}{c} X_{d}-X\\ Y_{d}-Y\\ \theta_{d}-\theta \end{array}\right]$$

(15) $$\left[\begin{array}{c} e_{x}\\ e_{y}\\ e_{\theta } \end{array}\right]=\left[\begin{array}{ccc} cos\theta & sin\theta & 0\\ -sin\theta & cos\theta & 0\\ 0 & 0 & 1 \end{array}\right]\left[\begin{array}{c} X_{d}-X\\ Y_{d}-Y\\ \theta_{d}-\theta \end{array}\right]$$

The outer controller can be designed as (16), where $\left(v_{rx}\;v_{ry}\;\omega_{r}\right)$ are the expect velocity, $\left(V_{xc}\;V_{yc}\;\omega_{c}\right)$ are the output of controller and $\left(k_{1}\;k_{2}\;k_{3}\right)$ are the adjustable parameters. Since $k_{1}$, $k_{2}$, and $k_{3}$ are the parameters of the outer loop proportional controller, they can be selected according to the constraints and control requirements of the actual system using the engineering tuning method.

(16) $$\left\{\begin{array}{c} V_{xc}=v_{rx}+k_{1}e_{x}\\ V_{yc}=v_{ry}+k_{2}e_{y}\\ \omega_{c}=\omega_{r}+k_{3}e_{\theta } \end{array}\right.$$

### 3.5. Preview Strategy

**Figure 5:**
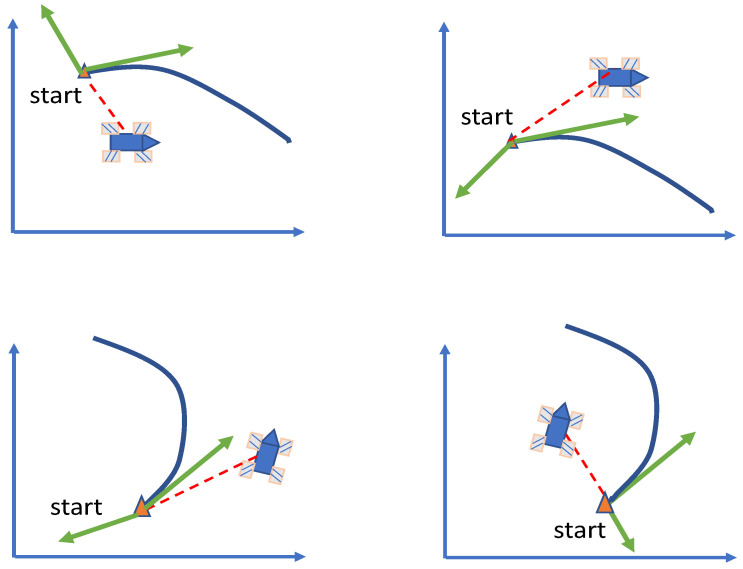
Track start lag.

In most robot application scenarios, the tracking trajectory is determined. In some cases, the starting point of tracking may be unreasonable, as shown in Figure 5. The figure shows that the starting point of the trajectory lags behind the robot. In this case, the starting point of the trajectory should not be used as the starting point of the tracking. A more reasonable way is to enter into the trajectory at other points on the trajectory.

**Figure 6:**
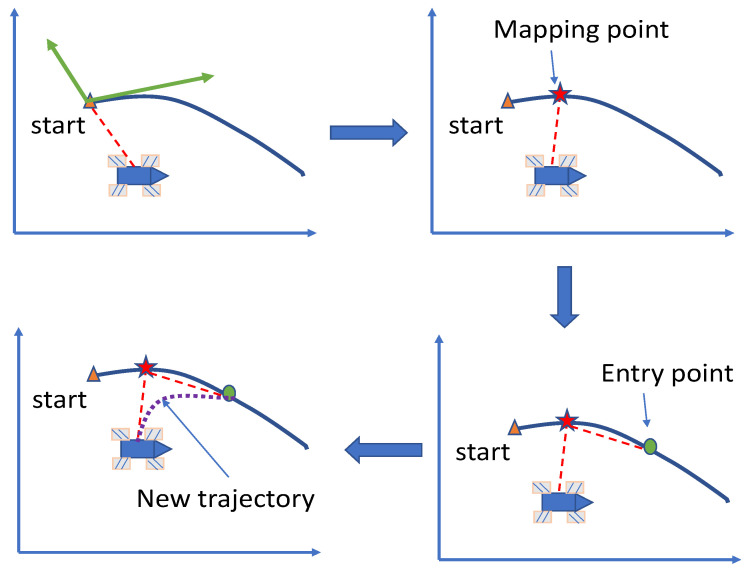
Sketch of the preview method.

To find the entry point, a PS is proposed. The flow of this method is shown in Figure 6.

Figure 6 reveals that the preview method consists of four parts. First, judge whether a preview is required according to the method shown in Figure 5, and then map the position of the robot to the trajectory to find the mapping point. Then find the entry point according to the distance between the robot and the mapping point. Finally, the trajectory is re planned according to the position and speed of the entry point. Since the re planned trajectory should be as close to the original trajectory as possible, polynomial interpolation cannot be used for planning.

The Bezier curve is a curve obtained by approximating the line segment of control points [26]. The mathematical expression is (17).

(17) $$P\left(t\right)=\sum_{i=0}^{n}\;P_{i}B_{i}^{n}\left(t\right)$$

where $P_{i}=\left[P_{0},P_{1}\ldots P_{n}\right]$ are the control points. $B_{i}^{n}\left(t\right)$ is the ith Bernstein polynomial of order *n*.

(18) $$\begin{array}{c} B_{i}^{n}\left(t\right)=C_{n}^{i}t^{i}{\left(1-t\right)}^{n-i}\\ C_{n}^{i}=\frac{n!}{i!(n-i)!} \end{array}$$

**Figure 7:**
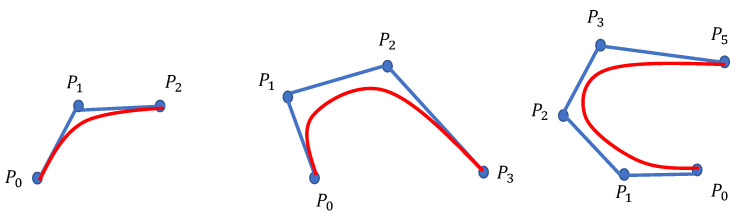
Bezier curve.

As shown in Figure 7, the Bezier curve can not only pass through the specified control points, but also its topology is similar to the control point segment.

Based on this feature, trajectory planning can be carried out through robot position, entry point and virtual control point. The obtained trajectory is shown in (19)

(19) $$\begin{array}{c} X_{ref}\left(t\right)={\left(1-\frac{t}{tf}\right)}^{2}x+2\left(1-\frac{t}{tf}\right)\frac{t}{tf}x_{vir}+{\left(\frac{t}{tf}\right)}^{2}x_{ety}\\ Y_{ref}\left(t\right)={\left(1-\frac{t}{tf}\right)}^{2}y+2\left(1-\frac{t}{tf}\right)\frac{t}{tf}y_{vir}+{\left(\frac{t}{tf}\right)}^{2}y_{ety} \end{array}$$

where tf is the end time, (x,y) is the position of the robot, $\left(x_{vir},y_{vir}\right)$ is the position of the virtual control point, and $\left(x_{ety},y_{ety}\right)$ is the position of the entry point,

According to (19), the velocity can be expressed as

(20) $$\begin{array}{c} {\dot{X}}_{ref}\left(t\right)=-2\left(\frac{1}{tf}-\frac{t}{tf^{2}}\right)x+\left(\frac{2}{tf}-\frac{4t}{tf^{2}}\right)x_{vir}+\frac{2t}{tf^{2}}x_{ety}\\ {\dot{y}}_{ref}\left(t\right)=-2\left(\frac{1}{tf}-\frac{t}{tf^{2}}\right)y+\left(\frac{2}{tf}-\frac{4t}{tf^{2}}\right)y_{vir}+\frac{2t}{tf^{2}}y_{ety} \end{array}$$

Since the speed at the end is required to be continuous, the virtual control point can be obtained as (21)

(21) $$\begin{array}{c} x_{vir}=x_{ety}-\frac{{\dot{X}}_{ref}\left(tf\right)tf}{2}\\ y_{vir}=y_{ety}-\frac{{\dot{Y}}_{ref}\left(tf\right)tf}{2} \end{array}$$

### 3.6. Stability Analysis

**Theorem** **1.** *Assume that the uncertain function $F\left(\omega \right)$ has a bound D, that is, $|F\left(\omega \right)|\leq D$. The approximation error $\tilde{F}\left(\omega \right)$ has a bound φ, that is, $|\tilde{F}\left(\omega \right)|\leq \varphi$. Then, the control scheme defined by (13), can guarantee that the system has the following properties:*

1. *All signals are bounded.*
2. *The tracking error and their derivatives converge to bounded when $\left.t\to \infty \right.$.*

**Proof of the Theorem.**  Set the following Lyapunov function.

(22) $$\begin{array}{cc} V & =\frac{1}{2}s^{T}Ms+\frac{1}{2}{\tilde{\theta }}^{T}\Gamma_{r}\tilde{\theta } \end{array}$$

where $\tilde{\theta }=\theta^{*}-\hat{\theta }$. the derivative of (22) is

(23) $$\begin{array}{cc} \dot{V} & =s^{T}M\dot{s}-{\tilde{\theta }}^{T}\Gamma_{r}\dot{\hat{\theta }} \end{array}$$

Applying (6), (23) can be rewritten as (24)

(24) $$\begin{array}{cc} \dot{V} & =s^{T}(F\left(\omega \right)-\hat{F}\left(\omega |\theta \right)-Hs)-{\tilde{\theta }}^{T}\Gamma_{r}\dot{\hat{\theta }} \end{array}$$

Applying (11) to get $F\left(\omega \right)=\theta^{*}\zeta \left(\omega \right)$ and $\hat{F}\left(\omega |\theta \right)=\hat{\theta }\zeta \left(\omega \right)$, (25) can be obtained.

(25) $$\begin{array}{cc} \tilde{F} & =F\left(\omega \right)-\hat{F}\left(\omega |\theta \right)=\tilde{\theta }\zeta \left(\omega \right)+\varphi \end{array}$$

Substituting (25) into (24), (26) can be obtained.

(26) $$\dot{V}=-s^{T}Hs+\tilde{\theta }\left(s\zeta \left(\omega \right)-\Gamma_{r}\dot{\hat{\theta }}\right)$$

and (27) can be obtained by introducing (12).

(27) $$\dot{V}=-s^{T}Hs+s^{T}\varphi$$

It can be seen from (27) that selecting the appropriate parameter *H* can make $\left|s^{T}\varphi \right|\leq s^{T}Hs$, so that $\dot{V}\leq 0$, the system is stable and the signal converges. □

## 4. Simulation

To verify the tracking performance of the designed control method, the trajectory tracking simulation of FM-OMR is carried out. The parameters of FM-OMR are shown in Table 3. In this paper, four SOT1FNNA are used to approximate the uncertain functions $F\left(\omega_{i}\right),i=1,2,3,4$.

### 4.1. Tracking Results

Given the reference trajectory equation as (28), and the uncertainty F(ω) as (29), the initial pose of FM-OMR and reference trajectory are $q\left(0\right)=\left(0,\;0,\;0\right)$, $\left(0.1,0.2,0\right)$, respectively.

(28) $$\left\{\begin{array}{c} x=0.1sin\left(0.5t\right)\\ y=0.2sin\left(t\right)\\ \theta =0 \end{array}\right.$$

(29) $$F\left(\omega \right)=\left[\begin{array}{c} 10\sin\left(\omega_{1}\right)+10t\\ 10\cos\left(\omega_{2}\right)+5t\\ 4\omega_{3}\\ 2\omega_{4} \end{array}\right]$$

**Figure 8:**
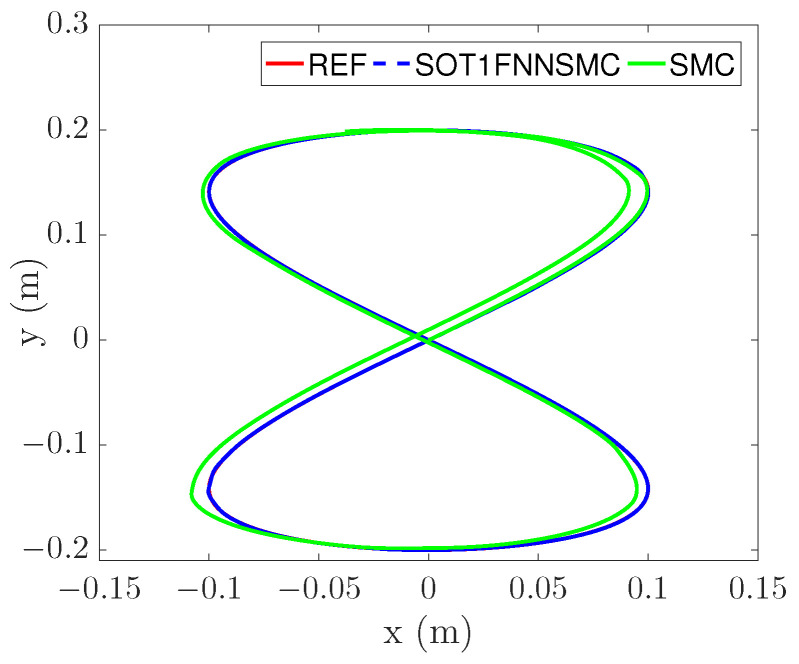
Figure-of-eight trajectory tracking.

The trajectory tracking results are shown in Figure 8. In the figure, “SMC” represents a common sliding mode controller. “SOT1FNNSMC” is the control scheme proposed in this paper. Figure 8 can clearly indicate that the tracking curve of SOT1FNNSMC is more consistent with the reference trajectory and has better tracking performance.

**Figure 9:**
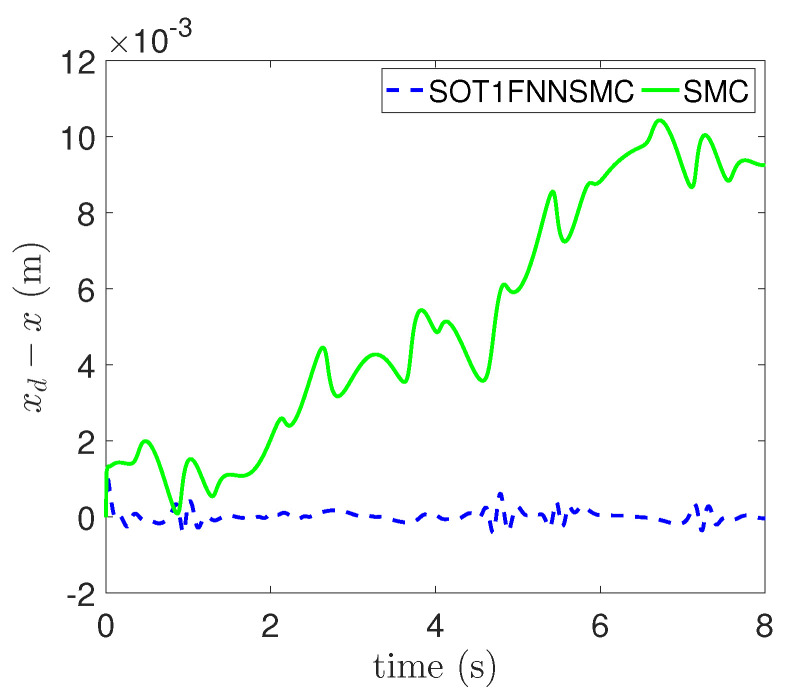
Tracking error of X component.

**Figure 10:**
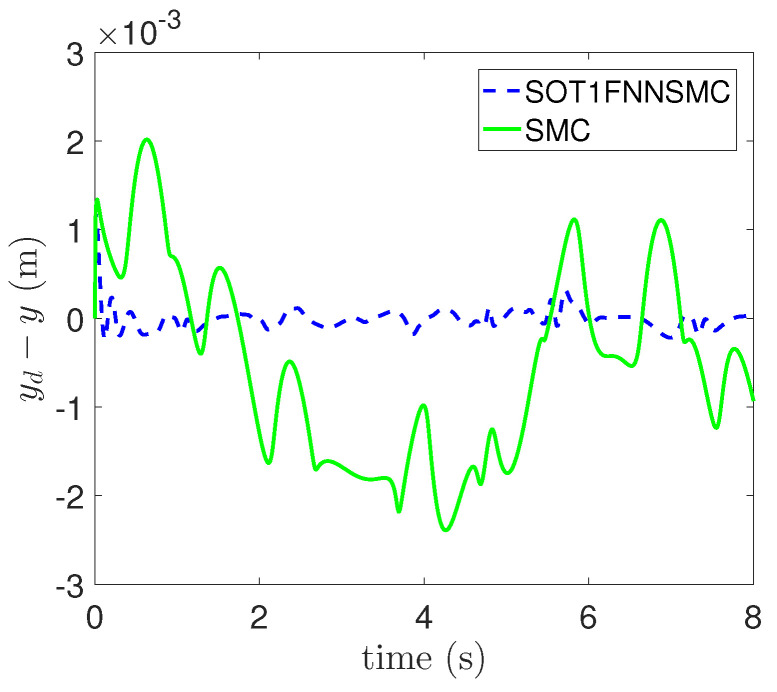
Tracking error of Y component.

**Figure 11:**
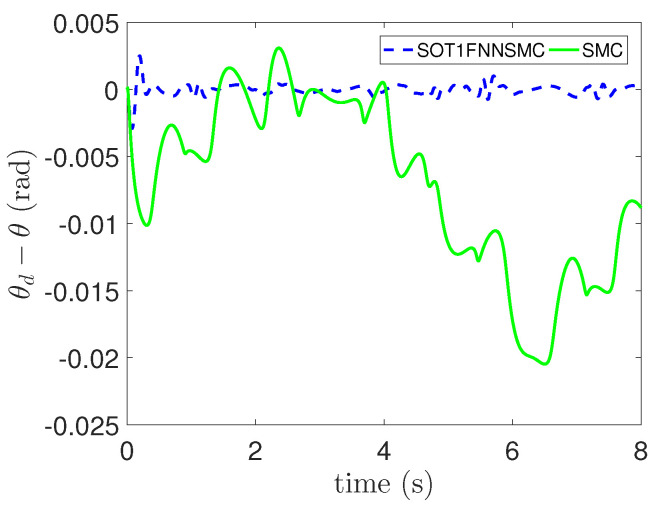
Tracking error of θ component.

In addition, the errors of each component of trajectory x, y and θ are respectively shown in Figure 9, Figure 10 and Figure 11. These figures clearly show that the tracking error of SOT1FNNSMC on each component is smaller and more stable than SMC. The results verify that the proposed method has good tracking performance.

**Figure 12:**
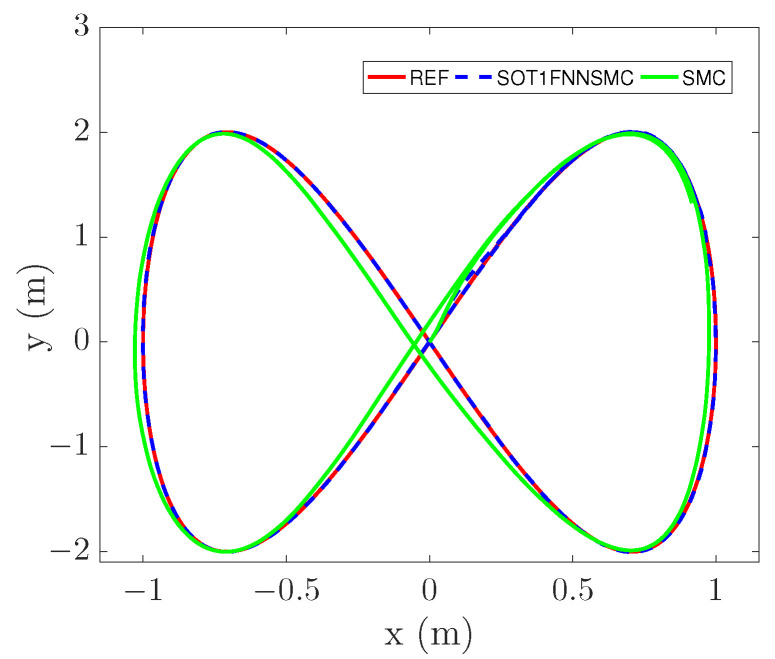
Figure-of-eight trajectory tracking-1.

In order to verify the effectiveness of this method in trajectory tracking of different ranges, a larger range of trajectory is selected, as shown in (30). The result is shown in Figure 12. It can be seen from the figure that even if the trajectory range increases, the robot can still complete the tracking well under the control of this method.

(30) $$\left\{\begin{array}{c} x=1sin\left(0.5t\right)\\ y=2sin\left(t\right)\\ \theta =0 \end{array}\right.$$

### 4.2. Approximation Performance

**Figure 13:**
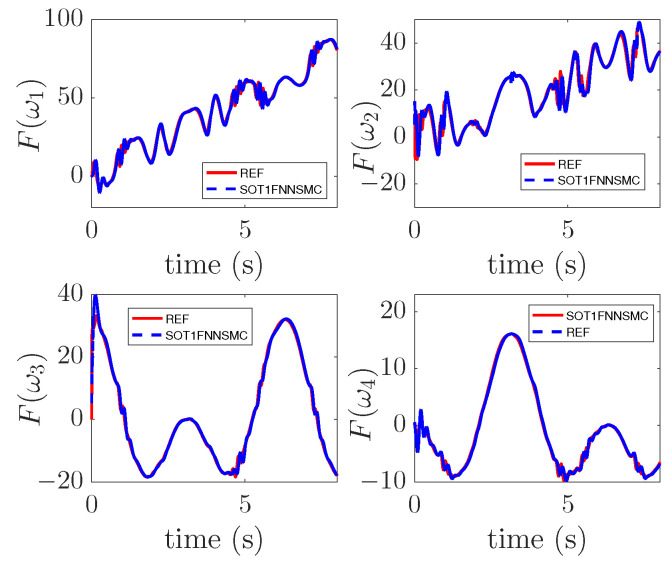
Approximation result.

The approximation result of SOT1FNNA to the uncertainty function is shown in Figure 13. The red curve in the figure is the uncertainty introduced into the model, and the blue curve is the output result of SOT1FNNA. The figure shows that the output curve of the network has a high coincidence with the uncertainty curve, which indicates that SOT1FNNA has a strong approximation ability. Although there are some fluctuations in the output of SOTIFNNA, it has little impact on the system, so it is generally acceptable.

### 4.3. Effect of Self-Organizing Algorithm

**Figure 14:**
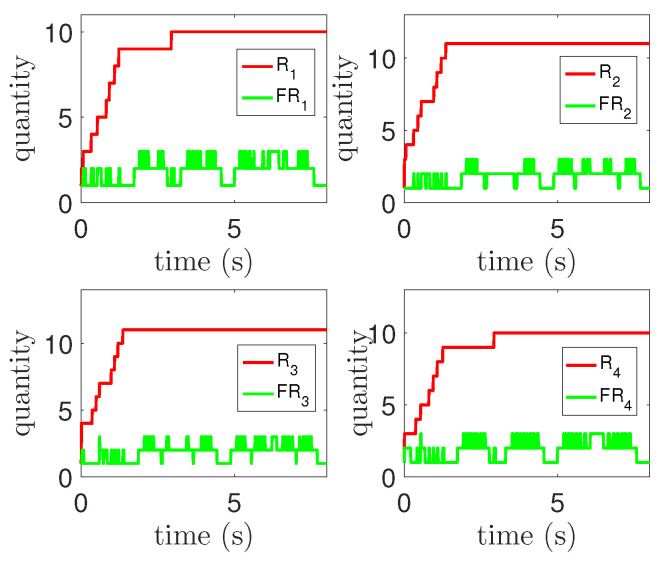
Rule number curve.

The self-organizing algorithm mainly aims at the problem of limited input caused by network presets. Therefore, the growth of rules can reflect whether the self-organizing algorithm has played a role. In addition, the self-organizing algorithm includes restrictions on access to the rule base to speed up the access process. The curve with the number of rules recorded in the simulation is shown in Figure 14, where $R_{i}$ represents the total number of rules and $FR_{i}$ represents the number of firing rules. It can be seen from the figure that the number of rules keeps growing over time and eventually tends to be stable. This result shows that when the existing rules cannot meet the threshold conditions, new rules are generated to correspond to them. With the stability of the tracking process, the number of rules tends to be stable. In addition, the number of firing rules is less than the total number of rules, which indicates that the restriction on rule access in the self-organizing algorithm can speed up the calculation process of output to a certain extent.

### 4.4. Preview Effect

To verify the effect of PS, two trajectories are set as shown in (31) and (32).

(31) $$\left\{\begin{array}{c} x=t\\ y=-0.25sin(t)+2\\ \theta =0 \end{array}\right.$$

(32) $$\left\{\begin{array}{c} x=0.3sin(t)-0.2\\ y=0.3sos(t)-0.1\\ \theta =0 \end{array}\right.$$

**Figure 15:**
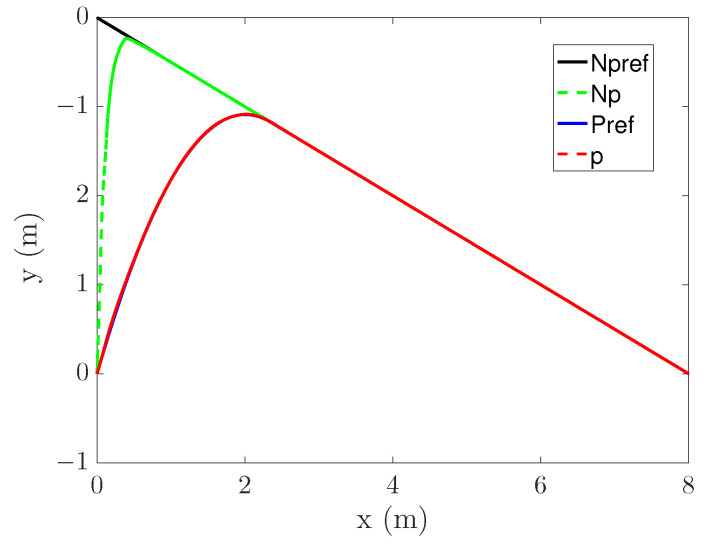
Line tracking.

**Figure 16:**
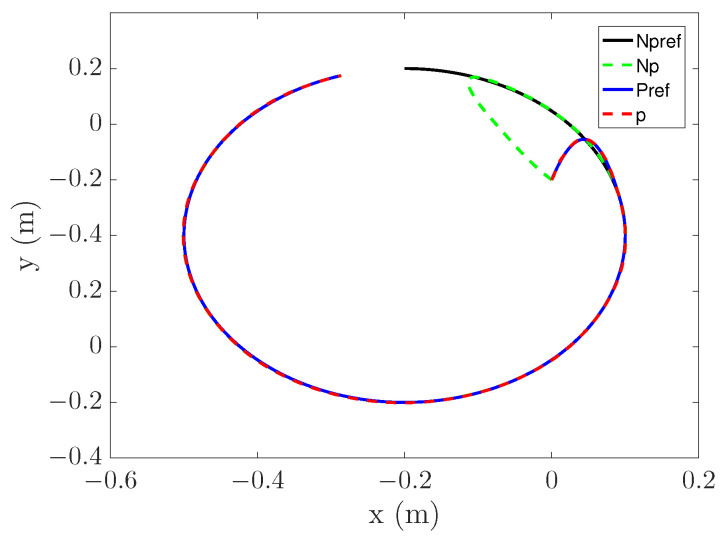
Circular trajectory tracking.

The tracking process is shown in Figure 15 and Figure 16, where Npref represents the reference trajectory without PS, Np represents the tracking curve without PS, Pref represents the reference trajectory with PS, and *p* represents the tracking curve with PS.

It can be seen from Figure 15 that the Np curve is highly curved, and even presents a certain angle. This is because there is no PS and only errors are considered. In contrast, the bending degree of the p curve is small, and the process of entering the original trajectory is smoother. It shows that the PS can optimize the starting point of tracking, thus making the tracking process reasonable. It can be seen from Figure 16 that the p curve is also better than Np. However, the p curve did not achieve excellent results. This is related to the selection of the entry point. By increasing the distance between the entry point and the mapping point, the trajectory after re-planning can be more stable.

## 5. Conclusions

A trajectory tracking control scheme based on SOT1FNNA and PS is proposed in this paper. Firstly, the SOTIFNNA in this scheme effectively estimates the uncertainties existing in the model, and improves the tracking accuracy compared with SMC. Secondly, the self-organizing algorithm based on rule growth effectively solves the problem of input constraints caused by network structure presetting. In addition, PS solves the problem of tracking curve distortion caused by the lag of initial trajectory starting point through trajectory re-planning. Finally, the simulation verifies the effectiveness of the proposed control scheme. 

## Figures and Tables

**Table 1:** Definition of the parameters.

Parameter	Definition
*M*	Inertial matrix
$\dot{\omega }$	The angular acceleration
$\omega_{i}^{\prime }$	The speed and angular of the roller
$\mu_{i}$	The coefficient of viscous friction
τ	The input torque
$I_{\omega }$	The rotational inertia of the wheel
$I_{z}$	The rotational inertia of robot
*R*	The radius of wheel
$l_{x}$	Track width
$l_{y}$	Wheelbase
$m_{r}$	The quality of robot

**Table 2:** Algorithm variable definition.

Variable	Definition
$x_{1}$	Input
$Mt^{x_{1}}$	Membership degree threshold
$A_{i}$	Fuzzy set
$N_{x_{1}}$	Number of fuzzy sets
RB	Rule base
$M_{max}^{x_{1}}$	Maximum membership degree
Rtab1	Fuzzy set table
$\theta_{new}$	Fuzzy rule consequent parameter

**Table 3:** Physical parameters.

Parameters	
x= 0.16 m	y= 0.12 m
m= 5.5 kg	R= 0.05 m
$I_{Z}=$ 6.921 kg·m^2^	$D_{\omega }=1$

## Data Availability

Not applicable.
